# SERPINB6 Promotes Epithelial‐Mesenchymal Transition via PI3K/AKT/mTOR Signalling Pathway in Glioma

**DOI:** 10.1111/jcmm.70711

**Published:** 2025-07-15

**Authors:** Ding Wang, Haiyang Wang, Wenhao Zheng, Heng Wang, Wenhua Yu, Quan Du

**Affiliations:** ^1^ Department of Neurosurgery Affiliated Hangzhou First People's Hospital, School of Medicine, Westlake University Hangzhou Zhejiang People's Republic of China

**Keywords:** epithelial‐mesenchymal transition, glioma, PI3K/AKT/mTOR, SERPINB6

## Abstract

Glioma is the most common and highly invasive type of brain cancer in the central nervous system, characterised by a low survival rate and poor prognosis. The role of SERPINB6 has been proven crucial in programmed necrosis and cancer progression. However, its role in glioma has not yet been clearly defined. This study utilised bioinformatics methods and in vitro and in vivo experiments to assess the expression, function and potential mechanisms of SERPINB6 in the development of glioma. Our study found that SERPINB6 plays a carcinogenic role in glioma, and its expression level is significantly negatively correlated with patient prognosis. The study also found that inhibiting SERPINB6 expression can hinder the epithelial‐mesenchymal transition (EMT) of glioma cells. Conversely, overexpression of SERPINB6 aggravated the occurrence of EMT. Particularly noteworthy is the mechanism of SERPINB6 in promoting EMT in glioma, which is achieved through the activation of the PI3K/AKT/mTOR signalling pathway. By activating the PI3K/AKT/mTOR pathway, SERPINB6 promotes EMT in glioma, demonstrating its great potential as a new target for glioma treatment.

## 1 Introduction

Glioma, especially glioblastoma multiforme (GBM), is the most common and highly malignant type of brain cancer in the central nervous system [1]. Classified as a grade IV glioma by the World Health Organisation, even with comprehensive treatment methods such as surgical resection, radiotherapy and chemotherapy, the average survival period for patients typically does not exceed 15 months [2]. This unfavourable prognosis underscores the urgent need to identify early diagnostic biomarkers and to deeply understand the molecular mechanisms of glioma progression.

Human serine protease inhibitor, clade B, member 6 (SERPINB6), a placental thrombin inhibitor, is widely expressed in various cell types, such as epithelial cells, monocytes and neutrophils [3]. Studies have found that SERPINB6 can inhibit cathepsin G and activate caspase 7 (a pro‐apoptotic protease), which helps prevent programmed necrosis in neutrophils and monocytes [4]. Recent research indicates that elevated SERPINB6 is associated with the development of hepatocellular carcinoma (HCC) and may play a role in advanced proliferation, migration and antioxidant stress resistance of colorectal cancer cells [5]. Additionally, SERPINB6 has been proposed as a prognostic indicator for cervical cancer, further suggesting its potential role in tumour growth and progression [6]. However, existing studies on SERPINB6 have predominantly focused on peripheral cancers, with limited exploration of its role in central nervous system malignancies, particularly glioma. Critically, no prior study has investigated whether SERPINB6 modulates EMT in glioma through the PI3K/AKT/mTOR signalling pathway—a key driver of glioma. This knowledge gap underscores the need to define the unique functional and molecular contributions of SERPINB6 in glioma pathogenesis.

EMT is a process involving the transformation of epithelium into a mesenchymal, characterised by the reduced intercellular adhesion and enhanced migratory and invasive capabilities [7]. This process is marked by the reduction of epithelial markers and the increase of mesenchymal markers. The PI3K/AKT/mTOR pathway is instrumental in EMT in malignant tumours, aiding in the transition to a more aggressive phenotype [8], which is essential in aspects such as cell survival, growth, proliferation, angiogenesis, transcription, translation and metabolism [9]. Particularly in the EMT process of malignant tumours, PI3K/AKT/mTOR signalling is instrumental in promoting tumour anti‐apoptosis and metastasis [10, 11].

In our study, SERPINB6 is highly upregulated in tumour samples and cells of glioma patients. Our research further demonstrates that the upregulation of SERPINB6 promotes the malignant characteristics of glioma in both in vivo and in vitro models. Most critically, SERPINB6 induces EMT and enhances the malignancy of glioma by activating the PI3K/AKT/mTOR pathway, suggesting its potential as a target in glioma treatment intervention.

## 2 Methods

### 2.1 Bioinformatics Analysis

Clinical data and transcriptome information of glioma patients were extracted from the databases of UCSC Xena (https://xenabrowser.net/datapages/) and CGGA (http://cgga.org.cn/download.jsp). Samples were grouped based on SERPINB6 expression; a survival analysis was conducted. Enrichment analysis was executed using the GSEA website. All charts and statistical analyses were completed by GraphPad Prism.

### 2.2 Clinical Sample Collection and Cell Culture

With patient consent, glioma and control brain tissue samples were collected from the Affiliated Hangzhou First People's Hospital and stored in liquid nitrogen for subsequent qRT‐PCR and Western Blot. Human glioma cell lines (U87, SNB19, A172, U251) and normal astrocytes (NHAs) were provided by the National Collection of Authenticated Cell Cultures in Shanghai, China, and cultured in DMEM containing 10% FBS at 37°C in 5% CO_2_ conditions.

### 2.3 Lentivirus Transfection

Shanghai GenePharma provided SERPINB6 overexpressed lentivirus (LV‐SERPINB6), two types of SERPINB6‐short hairpin RNAs (shSERPINB6‐1 and shSERPINB6‐2), and control vectors. Lentivirus was transfected into U87 and SNB19 cells using polybrene according to the manufacturer's instructions. After growing cells in a 24‐well plate to 60%–70% confluency, transfection was carried out, and the medium was changed 48 h after transfection. To obtain a stable cell line, the cells were cultured with medium including 5 μg/mL puromycin for 2 weeks, and qRT‐PCR and western blot were used to detect transfection efficiency.

### 2.4 qRT‐PCR Analysis

Total RNA was extracted using Invitrogen's Trizol reagent (15596018) and reverse transcribed into cDNA using a kit (K1691) provided by Thermo Fisher Scientific. qPCR analysis was conducted using SYBR Green provided by Vazyme, and gene expression was quantified using 2^−ΔΔCT^ methods. Primers used included forward and reverse primers for SERPINB6, and GAPDH as an internal reference. SERPINB6: Forward 5′‐ATGATGCGGTGTGCCAGATTCG‐3′, Reverse 5′‐TTGGTCTTGCTGTGCTGGATGAAG‐3′; GAPDH: Forward 5′‐TCATTGACCTCAACTACATGGTTT‐3′, Reverse 5′‐GAAGATGGTGATGGGATTTC‐3′.

### 2.5 Colony Formation

Cells were seeded in 6‐well plates at approximately 600 cells/well and cultured for 15 days. After washing the culture plates with PBS (HyClone, SH30256), cells cultured in plates were fixed and stained with 1% crystal violet to observe clone formation.

### 2.6 Western Blot

Protein samples were prepared using RIPA containing 1% protease inhibitor and quantified using the BCA method. After mixing the samples with loading buffer and heating at 100°C for 10 min, they were subjected to SDS‐PAGE electrophoresis and transferred to a PVDF membrane (Millipore, SEQ00010). The membrane was blocked with 5% BSA for 1 h, then incubated with primary and secondary antibodies at 4°C and room temperature, respectively. Protein expression was detected using the ECL method. The primary antibodies, including those against SERPINB6 (1:1200, Proteintech, 14962‐1‐AP), GAPDH (1:1800, Proteintech, 60004‐1‐Ig), E‐cadherin (1:1200, Proteintech, 20874‐1‐AP), N‐cadherin (1:800, Abcam, ab76011), Vimentin (1:1200, CST, 5741), p‐PI3K (1:800, Abcam, ab302958), PI3K (1:1200, CST, 4249), p‐AKT (1:800, CST, 9271), AKT (1:1200, CST, 4060), p‐mTOR (1:800, CST, 5536) and mTOR (1:1200, CST, 2983).

### 2.7 EdU Assay

Cells, cultured in confocal plates, were incubated with EdU solution (RiboBio, C10310‐1) for 2 h. Followed by fixation, cells were stained with Apollo Dye for 30 min in the dark, followed by DAPI staining. Images were obtained with a fluorescence microscope. The EdU‐positive cells were counted and analysed using ImageJ software.

### 2.8 Transwell Assay

The chambers were pre‐coated with Matrigel (DMEM 1:8 dilution, Corning) for 2 h for stabilisation. 5 × 10^4^ cells were seeded in each upper chamber with serum‐free DMEM (HyClone, SH30243). DMEM containing 20% FBS (BI, 04‐001) was placed in each lower chamber. After incubation for 48 h, cells were fixed and stained. Images of invasive cells were obtained under an optical microscope and analysed using Image J software.

### 2.9 Wound Healing Assay

Cells were cultured in 6‐well plates and, upon reaching 90%–100% confluency, a wound was created with 200 μL pipette tips, followed by culture with serum‐free DMEM to observe cell migration. Wound images were gotten with a microscope after 36 h, and the wound healing area was analysed using ImageJ software.

### 2.10 Xenograft Tumour Experiment

25 Balb/c nude mice, aged 4–6 weeks, were randomly grouped and housed under SPF conditions at the animal centre. 1 × 10^6^ U87 cells (overexpressing or knocking down SERPINB6 and control group [5 mice per group]) were subcutaneously injected into the right flank of each mouse. Tumour volume was measured using the formula V (mm^3^) = 0.5 × length (mm) × width^2^ (mm^2^).

### 2.11 Immunohistochemistry (IHC) and HE Staining

Xenograft tissues were fixed in 4% formaldehyde and embedded in paraffin. Sections were 5 μm thick, dewaxed, rehydrated and then treated in heat‐induced antigen retrieval solution. Sections were incubated with primary antibodies (Ki67: 1:200, ab15580 and Bax: 1:500, ab32503). Then sections were incubated with secondary antibodies for 1 h, followed by DAB (Beyotime, P0203) staining and counterstaining with haematoxylin. HE staining steps included dewaxing, rehydration, haematoxylin staining, hydrochloric acid ethanol differentiation, ammonia water bluing, eosin staining, followed by dehydration with graded alcohol, xylene clearing and mounting. IHC and HE stained images were captured using an optical microscope.

### 2.12 Statistical Methods

In vitro experiments were repeated 3 times, and in vivo experiments involved 5 mice per group. Data are presented as mean ± SD and were analysed with GraphPad Prism. Multiple group data were analysed with one‐way or two‐way ANOVA, and differences between two groups were assessed using Student's *t*‐test. Dunnett post hoc test is used to make statistical comparisons between the control and the experimental groups, and Tukey's post hoc test is used to compare experimental groups vs. experimental groups. All comparisons were made with similar variances among groups, and *p*‐value < 0.05 was considered statistically significant.

## 3 Results

### 3.1 Upregulation of SERPINB6 in Glioma and Its Clinical Significance

**FIGURE 1:**
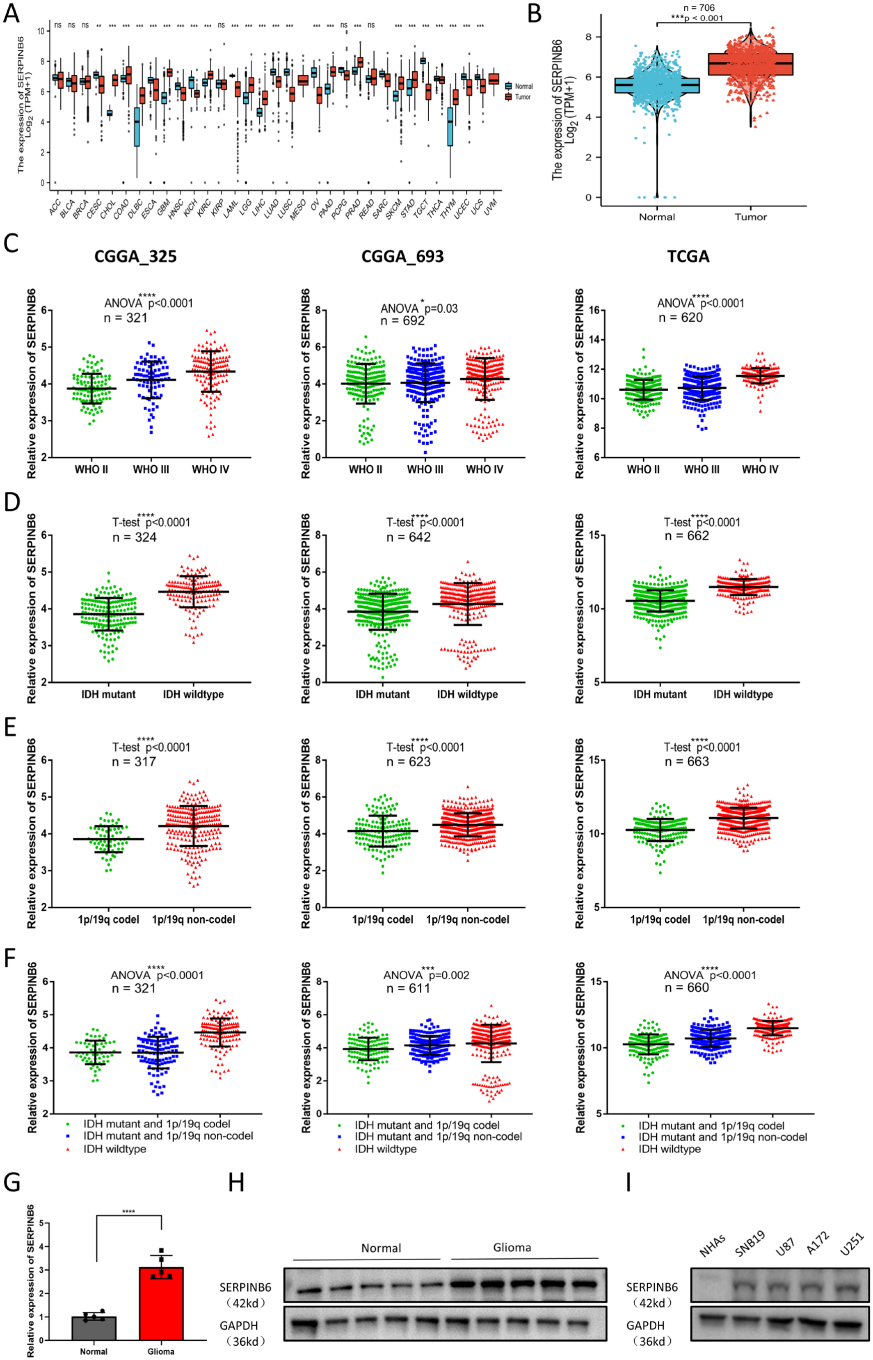
SERPINB6 is upregulated in glioma tissues and cell lines and predicts poor prognosis. (A) TCGA database analysis shows the SERPINB6 expression levels in 33 types of cancer tissues and their corresponding normal tissues. **p < 0.01, ***p < 0.001, Wilcoxon rank sum test. (B) TCGA database analysis shows the SERPINB6 expression levels in glioma tissues and their corresponding normal tissues. ***p < 0.001, Wilcoxon rank sum test. (C–F) SERPINB6 expression in different grades of glioma in TCGA, CGGA_693 and CGGA_693 datasets, respectively. *p < 0.05, **p < 0.01, ****p < 0.0001, one‐way ANOVA; ****p < 0.0001, Student's *t*‐test. (G) qRT‐PCR analysis of SERPINB6 expression in human glioma samples and normal brain tissues. **p < 0.01, Student's *t*‐test. (H) Immunoblot analysis of SERPINB6 expression in human glioma samples and normal brain tissues. GAPDH was used as loading control. (I) Immunoblot analysis of SERPINB6 expression in human glioma cell lines (U87, SNB19, A172 and U251) and Normal Human Astrocytes (NHAs). GAPDH was used as loading control.

To explore SERPINB6 expression in glioma, we initially analysed its expression in human cancer and normal tissues, utilising data from the TCGA & CGGA database. We observed that SERPINB6 mRNA was significantly upregulated in 12 out of 33 cancer types, including glioblastoma (GBM) and brain lower grade glioma (LGG), compared to normal tissues (Figure 1A). Specifically, SERPINB6 expression was markedly higher in glioma tissues relative to controls (Figure 1B). Further, we examined the relationship between SERPINB6 expression and various clinical characteristics in glioma. Utilising WHO classification, and considering isocitrate dehydrogenase (IDH) mutation and 1p/19q codeletion statuses, we observed distinct expression patterns. Higher‐grade gliomas, which are associated with poor prognosis, showed increased SERPINB6 expression compared to lower grades (Figure 1C). SERPINB6 levels were significantly higher in IDH wildtype subtypes compared to mutant subtypes (Figure 1D), and in 1p/19q non‐codeleted subtypes relative to codeleted subtypes (Figure 1E). Notably, in the IDH wildtype subgroup, SERPINB6 expression was higher compared to both the IDH mutant with 1p/19q codeletion and non‐codeletion subtypes (Figure 1F).

**FIGURE 2:**
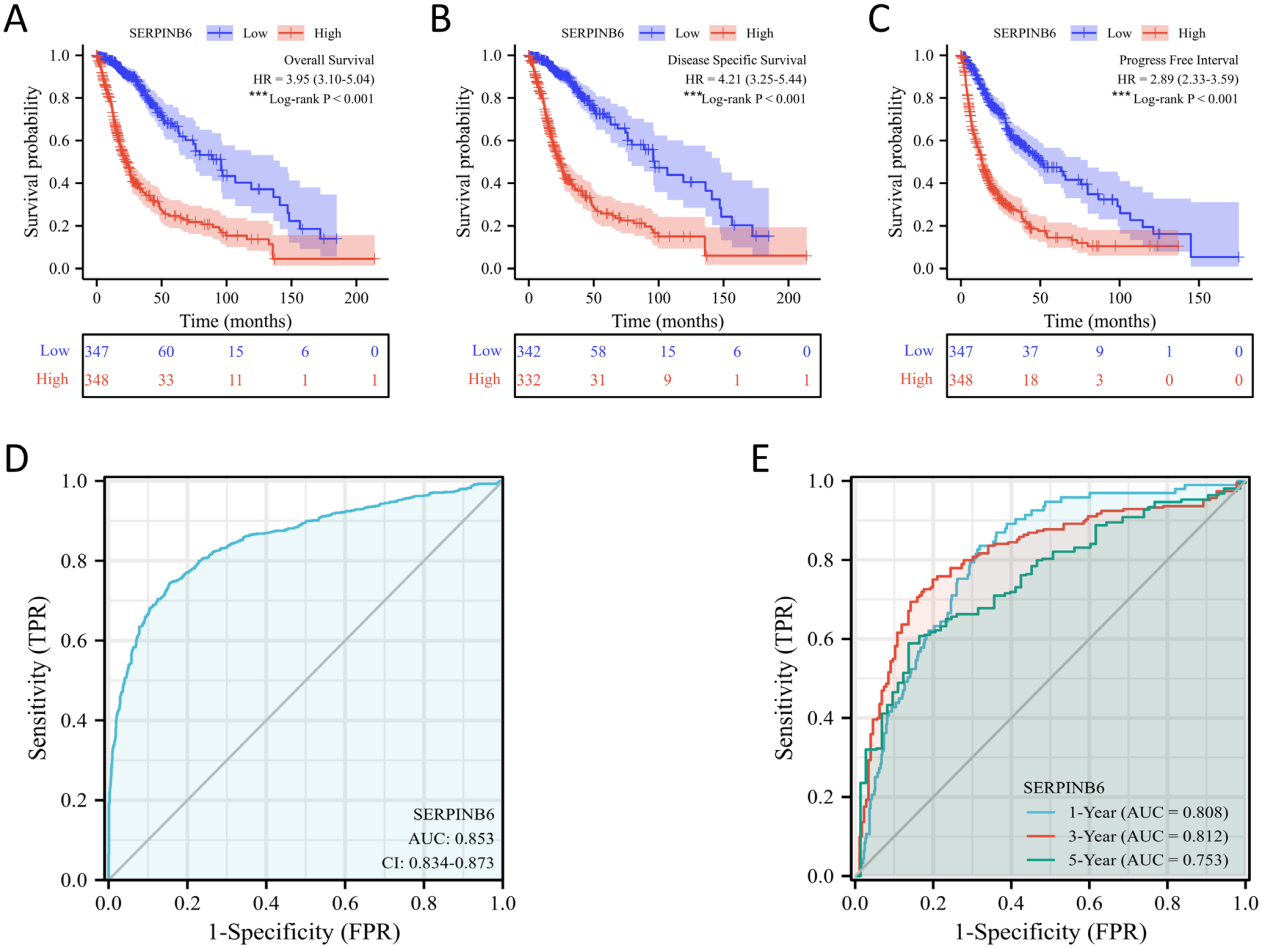
SERPINB6 shows a high prognostic prediction value in glioma patients. The Kaplan–Meier plotter database analysis shows the differences in (A) overall survival, (B) disease‐specific survival and (C) progression‐free interval of glioma patients with high‐ and low‐SERPINB6 expression levels. ***p < 0.001, Log‐rank test. (D) Diagnostic ROC curves to distinguish glioma tissues and normal tissues based on the SERPINB6 expression levels. (E) Time‐dependent survival ROC curves to predict 1‐, 3‐ and 5‐year survival rates of glioma patients based on the SERPINB6 expression levels.

To evaluate the clinical relevance of SERPINB6 in glioma, we conducted qRT‐PCR and western blot assays on five pairs of clinical glioma and normal tissues. The results indicated a significant elevation of SERPINB6 in glioma tissues (Figure 1G,H), a finding consistent with increased expression in glioma cell lines (SNB19, U87, A172, U251) compared to normal human astrocytes (NHAs) (Figure 1I). Kaplan–Meier survival analysis further revealed that high SERPINB6 expression correlated with lower overall survival (OS), disease‐specific survival (DSS), and progression‐free interval (PFI) in glioma patients (Figure 2A–C). The diagnostic value of SERPINB6 was affirmed by ROC and time‐dependent ROC curve analyses, demonstrating moderate accuracy (AUC > 0.7) in glioma prediction (Figure 2D,E).

In summary, our findings indicate that SERPINB6 is upregulated in glioma and is closely associated with established prognostic and diagnostic markers. Consequently, SERPINB6 emerges as a potential candidate for further functional and molecular investigations in both in vitro and in vivo settings.

### 3.2 Analysis of the Correlation Between SERPINB6 Expression and Immune Cell Infiltration

**FIGURE 3:**
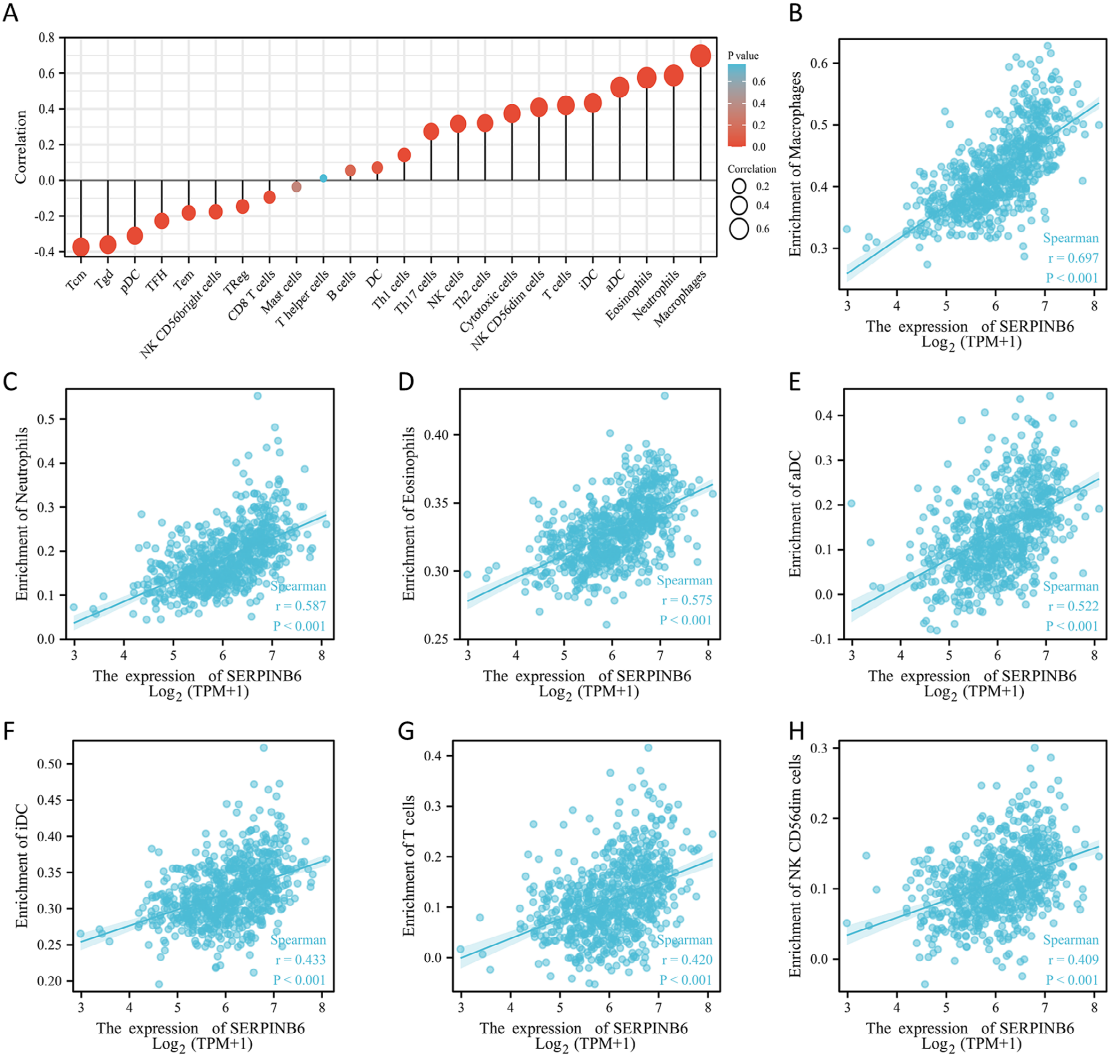
Analysis of the correlation between SERPINB6 expression and immune cell infiltration. (A) Correlation between various immune cell infiltrations in glioma tissues and SERPINB6 expression. (B–H) Correlation between SERPINB6 expression and the infiltration level of macrophages, neutrophils, eosinophils, aDC, iDC, T cells and NK CD56dim cells.

Besides tumour cells, there are various stromal cells, including fibroblasts, endothelial cells and immune cells, etc., which together constitute the tumour microenvironment. The type and degree of immune cell infiltration in the tumour microenvironment play an important role in tumour progression. Therefore, it is necessary to conduct quantitative studies on the types of immune cells in tumour tissues. Since the enrichment analysis found that SERPINB6 might be involved in the immune response of gliomas, we examined the relationship between SERPINB6 expression and immune cell infiltration levels through ssGSEA analysis. The correlations between various immune cell infiltrations in glioma tissues and SERPINB6 expression are shown in Figure 3A. Immune cells that are strongly positively correlated with SERPINB6 expression include macrophages (*r* = 0.697, *p* < 0.001, Figure 3B), neutrophils (*r* = 0.587, *p* < 0.001, Figure 3C), eosinophils (*r* = 0.575, *p* < 0.001, Figure 3D), activated dendritic cells (aDC, *r* = 0.522, *p* < 0.001, Figure 3E), immature dendritic cells (iDC, *r* = 0.433, *p* < 0.001, Figure 3F), T cells (*r* = 0.420, *p* < 0.001, Figure 3G), and CD56dim natural killer cells (*r* = 0.409, *p* < 0.001, Figure 3H).

### 3.3 Silencing SERP Inhibits Proliferation at Invasion Migration In Vitro

**FIGURE 4:**
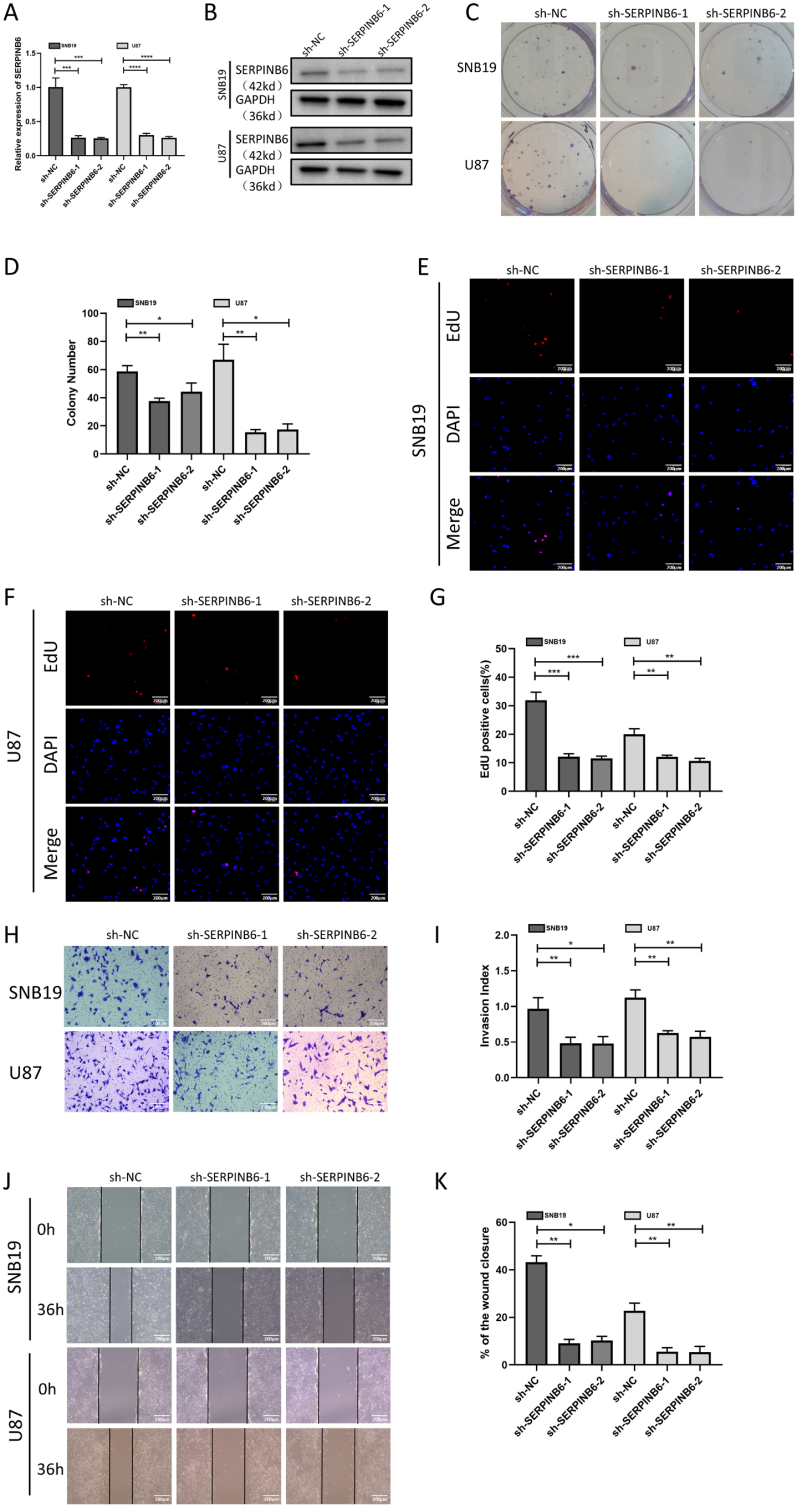
SERPINB6 silencing inhibits cell proliferation, migration and invasion in vitro. RT‐qPCR (A) and Immunoblot (B) analysis of SERPINB6 expression in U87 and SNB19 cells after transfection with the sh‐NC, sh‐SERPINB6‐1, or sh‐SERPINB6‐2. GAPDH was used as the loading control. ***p < 0.001 and ****p < 0.0001, one‐way ANOVA. (C, D) Colony formation capacity of sh‐NC, sh‐SERPINB6‐1 and sh‐SERPINB6‐2 groups in U87 and SNB19 cells. *p < 0.05 and **p < 0.01, one‐way ANOVA. (E–G) EdU assay was applied to compare the cell proliferation ability of sh‐NC, sh‐SERPINB6‐1 and sh‐SERPINB6‐2 groups in U87 and SNB19 cells. **p < 0.01 and ***p < 0.001, one‐way ANOVA. (H, I) Effect of SERPINB6 on cell invasion by the matrigel transwell assay. *p < 0.05 and **p < 0.01, one‐way ANOVA. (J, K) Effect of SERPINB6 on cell migration assessed by wound healing assay in 36 h. *p < 0.05 and **p < 0.01, one‐way ANOVA.

To elucidate the functional impact of SERPINB6 on glioma, we established stable SERPINB6 knockdown in SNB19 and U87 cells using siRNAs (shSERPINB6‐1 and shSERPINB6‐2). Subsequent qRT‐PCR and western blot assays confirmed a significant reduction in SERPINB6 levels in the knockdown groups compared to the negative control (Figure 4A,B). Colony‐forming assays revealed that SERPINB6 knockdown notably diminished the colony‐forming abilities of both SNB19 and U87 cells (Figure 4C,D). Furthermore, EdU assays indicated a substantial decrease in the proliferative capabilities of these cells post‐SERPINB6 silencing (Figure 4E–G). Additionally, wound healing and transwell assays demonstrated that SERPINB6 knockdown markedly impeded the migratory and invasive capacities of SNB19 and U87 cells (Figure 4H–K).

### 3.4 SERPINB6 Overexpression Enhances Proliferation, Invasion and Migration In Vitro

**FIGURE 5:**
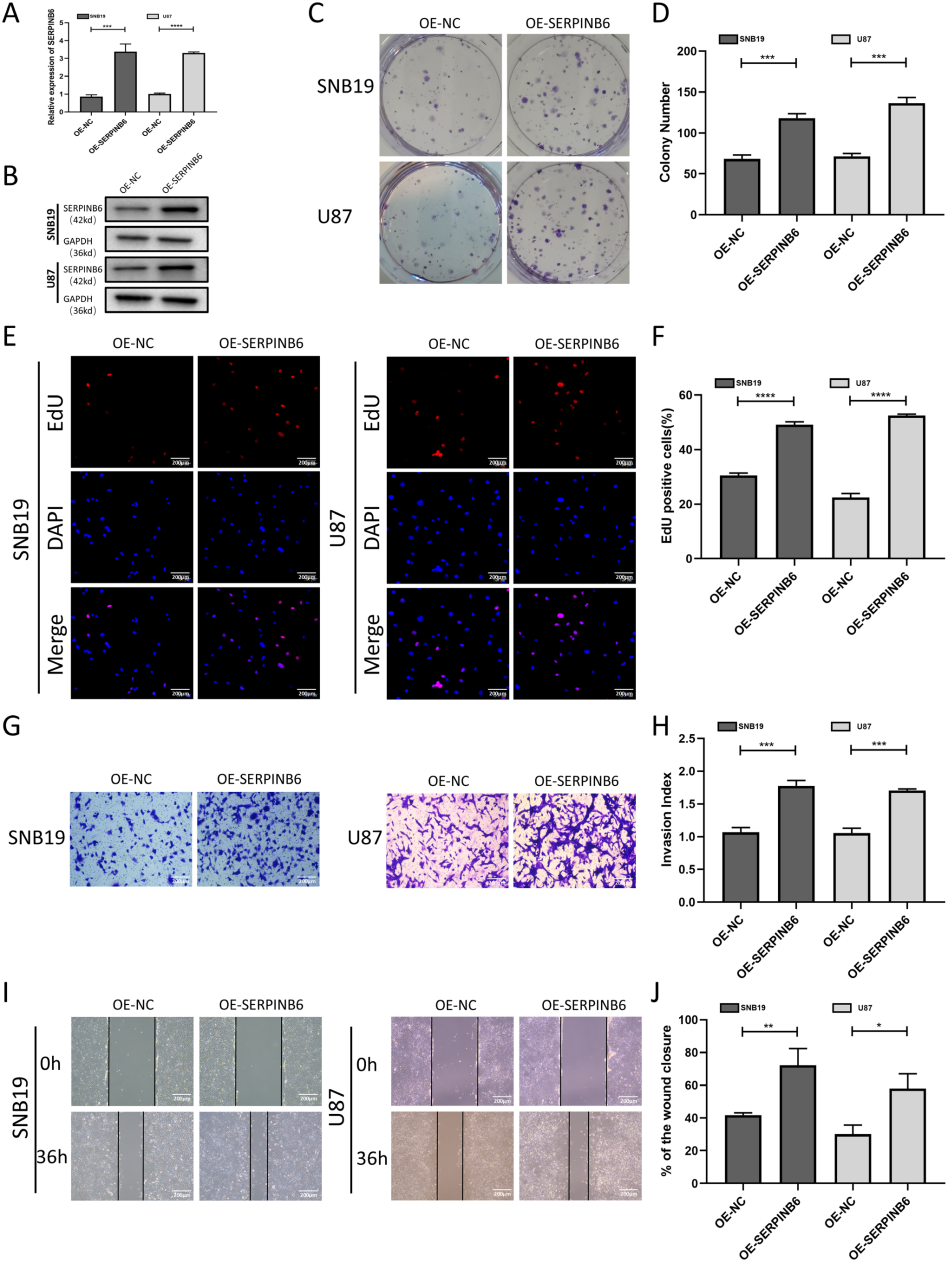
SERPINB6 overexpression promotes cell proliferation, migration and invasion in vitro. RT‐qPCR (A) and Immunoblot (B) analysis of SERPINB6 expression in U87 and SNB19 cells after transfection with the OE‐NC or OE‐SERPINB6. GAPDH was used as the loading control. ***p < 0.001 and ****p < 0.0001, Student's *t*‐test. (C, D) Colony formation capacity of OE‐NC and OE‐SERPINB6 groups in U87 and SNB19 cells. ***p < 0.001, Student's *t*‐test. (E, F) EdU assay was applied to compare the cell proliferation ability of OE‐NC and OE‐SERPINB6 groups in U87 and SNB19 cells. ****p < 0.0001, Student's *t*‐test. (G, H) Effect of SERPINB6 on cell invasion by the matrigel transwell assay. ***p < 0.001, Student's *t*‐test. (I, J) Effect of SERPINB6 on cell migration assessed by wound healing assay in 36 h. *p < 0.05 and **p < 0.01, Student's *t*‐test.

We next established stable SERPINB6 overexpression in SNB19 and U87 cells. qRT‐PCR and western blot assays verified a significant elevation in SERPINB6 expression in these overexpressing cells compared to the negative control (Figure 5A,B). Colony‐forming assays showed an increase in colony formation in cells transfected with the SERPINB6 overexpression vector (Figure 5C,D). Additionally, EdU assays confirmed that SERPINB6 overexpression significantly enhanced the proliferative capacity of both SNB19 and U87 cells (Figure 5E,F). Wound healing and transwell assays further revealed that SERPINB6 overexpression led to significant increases in the invasion and migration capabilities of these cells (Figure 5G–J).

### 3.5 SERPINB6 Promotes Glioma Growth In Vivo

**FIGURE 6:**
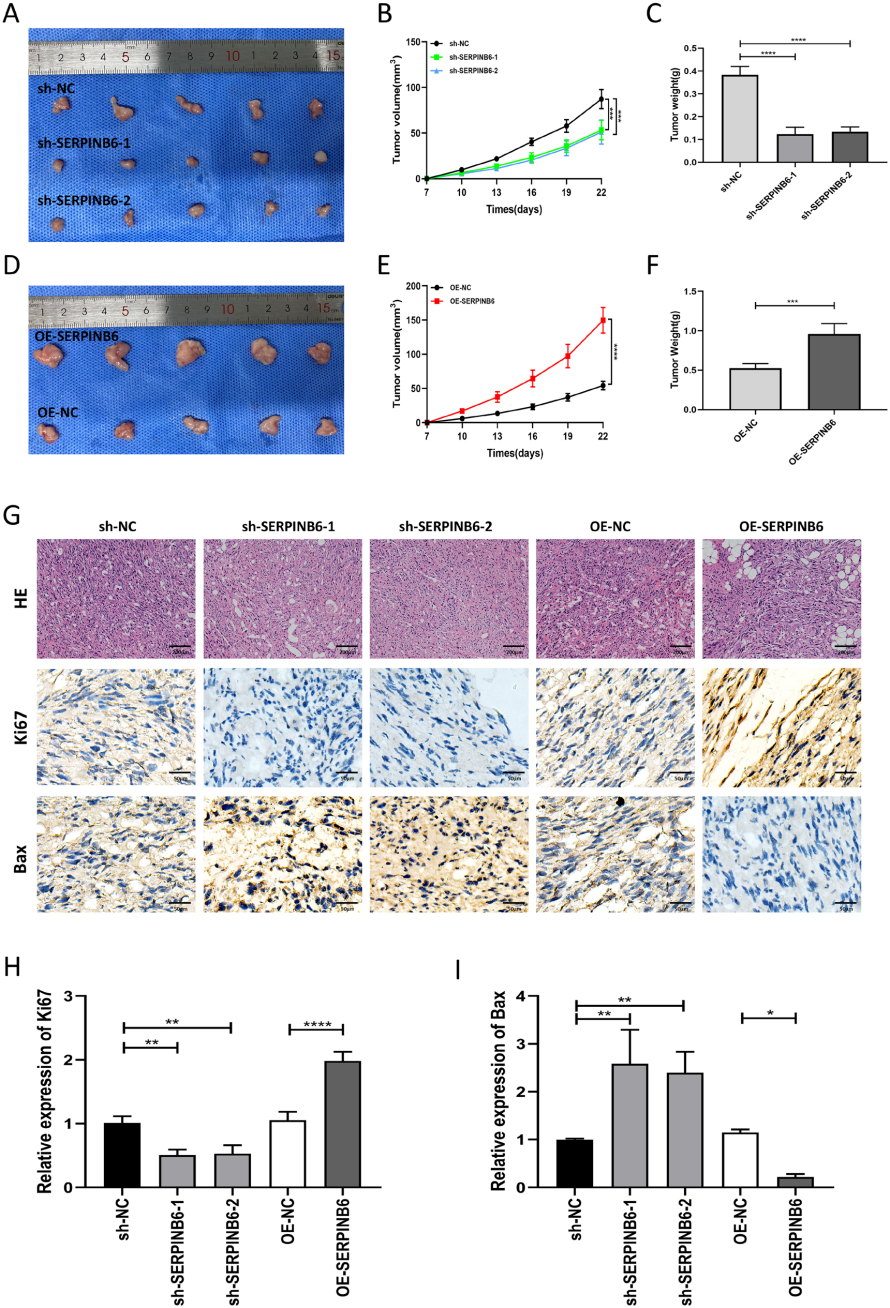
SERPINB6 promotes glioma growth in vivo. (A) Tumorigenicity assays were performed in nude mice following subcutaneous inoculation of SNB19 cells transfected with sh‐NC, sh‐SERPINB6‐1, or sh‐SERPINB6‐2. Tumour (B) volume and (C) weight were measured in the sh‐NC, sh‐SERPINB6‐1 and sh‐SERPINB6‐2 groups. ***p < 0.001, two‐way ANOVA; ****p < 0.0001, one‐way ANOVA. (D) Tumorigenicity assays were performed in nude mice after subcutaneous inoculation of SNB19 cells transfected with OE‐NC or OE‐SERPINB6. Tumour (E) volume and (F) weight were measured in the OE‐NC and OE‐SERPINB6 groups. ****p < 0.0001, two‐way ANOVA; ***p < 0.001, Student's *t*‐test. (G) H&E staining and IHC staining of Ki67 and Bax in sh‐NC, sh‐SERPINB6‐1, sh‐SERPINB6‐2, OE‐NC and OE‐SERPINB6 groups. (H, I) Relative expression of Ki67 and Bax in H&E staining, respectively. *p < 0.05, **p < 0.01, ****p < 0.0001.

To validate the pro‐tumorigenic effects of SERPINB6 in vivo, we conducted experiments in tumour‐bearing Balb/c nude mice. In line with our in vitro findings, SERPINB6 knockdown resulted in reduced tumour volume and weight compared to the control group (Figure 6A–C), whereas SERPINB6 overexpression led to increased tumour volume and weight (Figure 6D–F). Additionally, HE and IHC staining of xenograft tissues from these mice revealed that SERPINB6 knockdown significantly diminished Ki67 expression and increased Bax expression. In contrast, SERPINB6 overexpression produced the opposite effects (Figure 6G). Collectively, these findings indicate that SERPINB6 significantly enhances the tumorigenic potential of glioma.

### 3.6 SERPINB6 Promotes EMT via PI3K/AKT/mTOR Signalling Pathway

**FIGURE 7:**
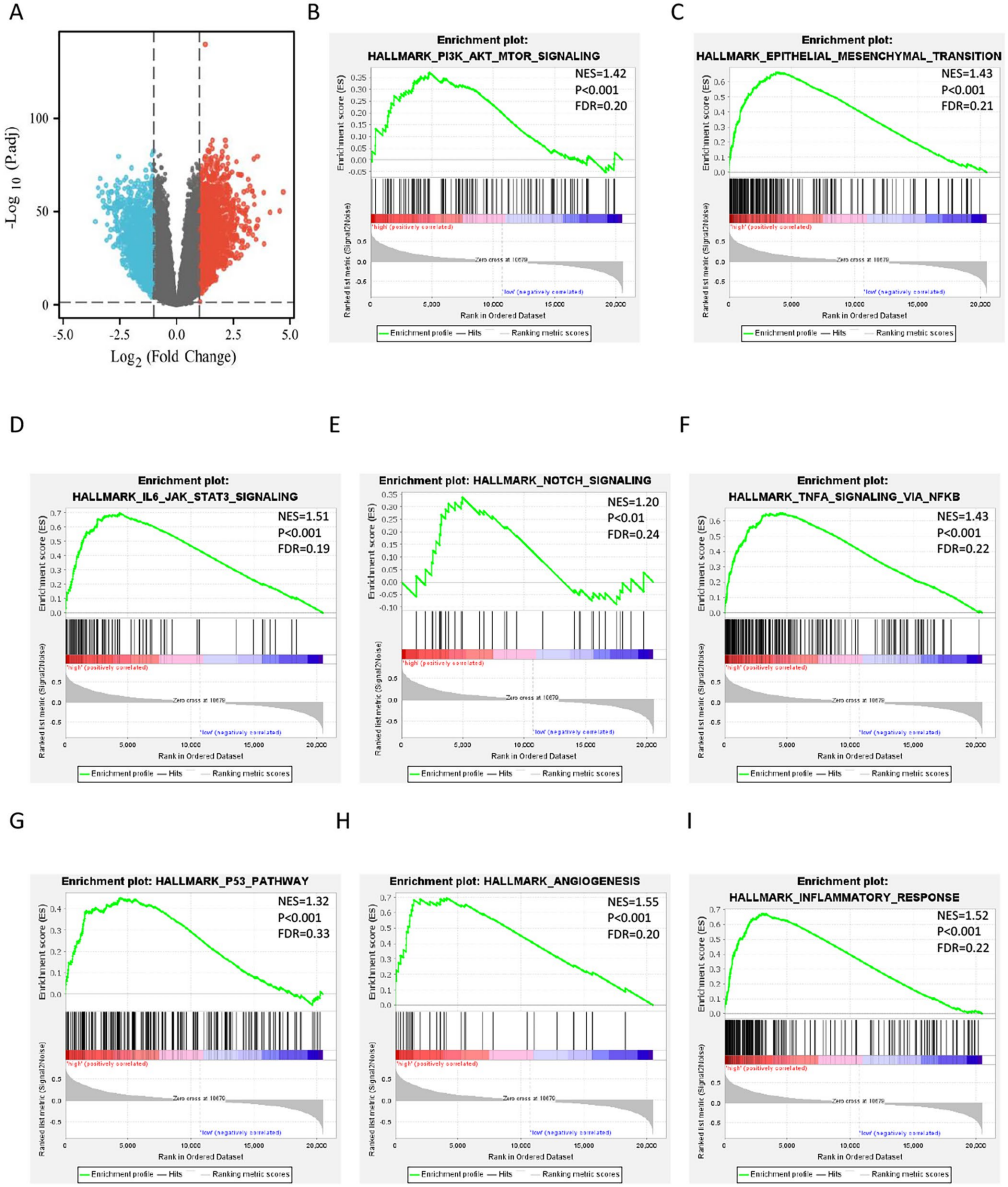
Functional enrichment analysis of the differentially expressed genes (DEGs) based on the SERPINB6 expression levels in glioma. (A) The volcano plots show the DEGs in the glioma patients with high‐ and low‐SERPINB6 expression. (B–I) Gene Set Enrichment Analysis (GSEA) of the altered signalling pathways in the glioma tissues based on the SERPINB6‐associated DEGs between the high‐ and low‐SERPINB6 expression groups in glioma.

To gain insight into the molecular mechanisms by which SERPINB6 mediates the malignant phenotype in glioma, we first identified differentially expressed genes (DEGs) between SERPINB6‐high and SERPINB6‐low expression samples using the limma package (Figure 7A). Gene Set Enrichment Analysis (GSEA) was then performed, revealing significant enrichment of several key pathways in SERPINB6‐high glioma patients, including PI3K/AKT/mTOR signalling, epithelial‐mesenchymal transition (EMT), IL‐6‐JAK‐STAT3 signalling, NOTCH signalling, TNFα signalling via NF‐κB, P53 pathway, angiogenesis and inflammatory response (Figure 7B–I), all of which are known to be involved in cancer progression.

**FIGURE 8:**
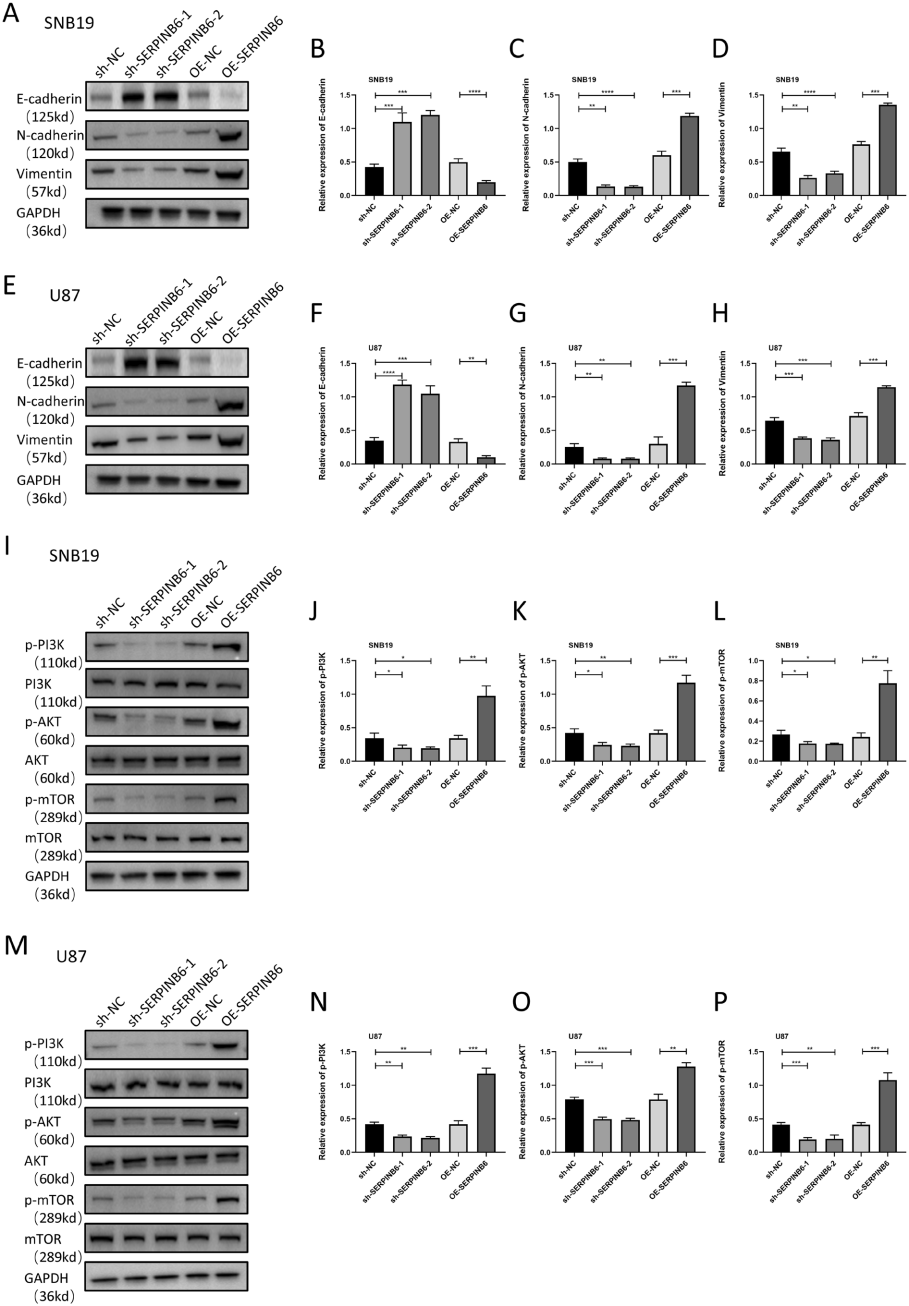
SERPINB6 promotes EMT and PI3K/AKT/mTOR signalling pathway. (A–H) Immunoblot analysis of E‐cadherin, N‐cadherin and Vimentin in U87 and SNB19 cells transfected with sh‐NC, sh‐SERPINB6‐1, sh‐SERPINB6‐2, OE‐NC and OE‐SERPINB6. GAPDH was used as loading control. **p < 0.01, ***p < 0.001 and ****p < 0.0001, one‐way ANOVA; Student's *t*‐test. (I–P) Immunoblot analysis of p‐PI3K, PI3K, p‐AKT, AKT, p‐mTOR and mTOR in U87 and SNB19 cells transfected with sh‐NC, sh‐SERPINB6‐1, sh‐SERPINB6‐2, OE‐NC and OE‐SERPINB6. GAPDH was used as loading control. *p < 0.05, **p < 0.01 and ***p < 0.001, one‐way ANOVA; Student's *t*‐test.

Given the enriched EMT pathway's known role in promoting cancer proliferation and invasion [12], we conducted Western blot assays to evaluate EMT marker levels. These assays revealed that SERPINB6 knockdown increased the expression of epithelial markers like E‐cadherin and decreased mesenchymal markers such as N‐cadherin and Vimentin (Figure 8A–H). Conversely, SERPINB6 overexpression produced the opposite effect, indicating that SERPINB6 is a key driver of EMT in glioma.

Furthermore, considering the significant activation of the PI3K/AKT/mTOR pathway in SERPINB6‐high patients and the role of EMT as a downstream effector of this pathway [13], we assessed the expression changes of p‐PI3K, PI3K, p‐AKT, AKT, p‐mTOR and mTOR. The results demonstrated that SERPINB6 overexpression upregulated the phosphorylation of PI3K, AKT and mTOR, while its knockdown suppressed their protein levels (Figure 8I–P). These findings suggest that SERPINB6 activates the PI3K/AKT/mTOR signalling pathway, potentially serving as an upstream factor of EMT in glioma.

### 3.7 SERPINB6 Enhances Glioma Malignancy via the EMT Process Activated by the PI3K/AKT/mTOR Pathway

**FIGURE 9:**
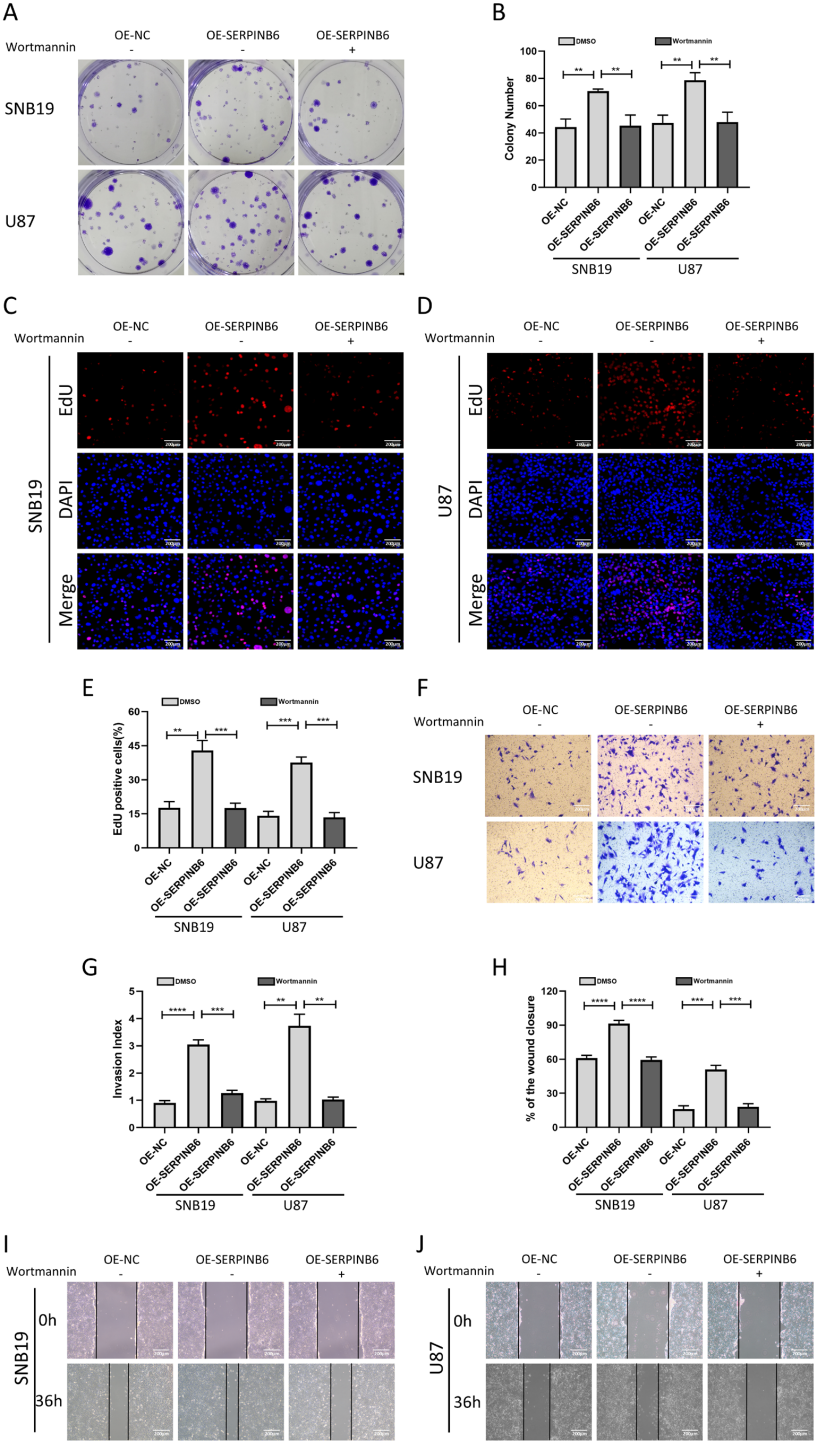
Inhibiting PI3K/AKT/mTOR signalling retards proliferation, invasion and migration of glioma cells induced by SERPINB6 overexpression. (A, B) Colony formation capacity of OE‐NC, OE‐SERPINB6 and OE‐SERPINB6 combined with Wortmannin groups in U87 and SNB19 cells. **p < 0.01, one‐way ANOVA. (C–E) EdU assay was applied to compare the cell proliferation ability of OE‐NC, OE‐SERPINB6 and OE‐SERPINB6 combined with Wortmannin groups in U87 and SNB19 cells. **p < 0.01 and ***p < 0.001, one‐way ANOVA. (F, G) Transwell assay assessed the cell invasion capacity of OE‐NC, OE‐SERPINB6 and OE‐SERPINB6 combined with Wortmannin groups in U87 and SNB19 cells. **p < 0.01, ***p < 0.001 and ****p < 0.0001, one‐way ANOVA. (H–J) Wound healing assay assessed the cell migration capacity of OE‐NC, OE‐SERPINB6 and OE‐SERPINB6 combined with Wortmannin groups in U87 and SNB19 cells. ***p < 0.001 and ****p < 0.0001, one‐way ANOVA.

We conducted a series of assays to examine the proliferative, invasive and migratory capacities of SNB19 and U87 glioma cells. Colony‐forming and EdU assays were first employed to assess cell proliferation. Our results demonstrated that inhibiting PI3K with the small‐molecule inhibitor Wortmannin (50 nM, treated in vitro for 24 h) significantly reduced cell proliferation induced by SERPINB6 overexpression (Figure 9A–E). This finding aligns with our hypothesis regarding the role of the PI3K/AKT/mTOR pathway in SERPINB6‐mediated malignancy.

To further evaluate the invasive capacities of these cells, Transwell assays were performed. We observed that PI3K inhibition notably impaired the cells' ability to penetrate the matrix gel, a phenomenon previously enhanced by SERPINB6 overexpression (Figure 9F,G). Additionally, wound healing assays were conducted to examine cell migration. The results indicated that PI3K inhibition significantly impeded cell migration, which had been augmented by SERPINB6 overexpression (Figure 9H–J).

**FIGURE 10:**
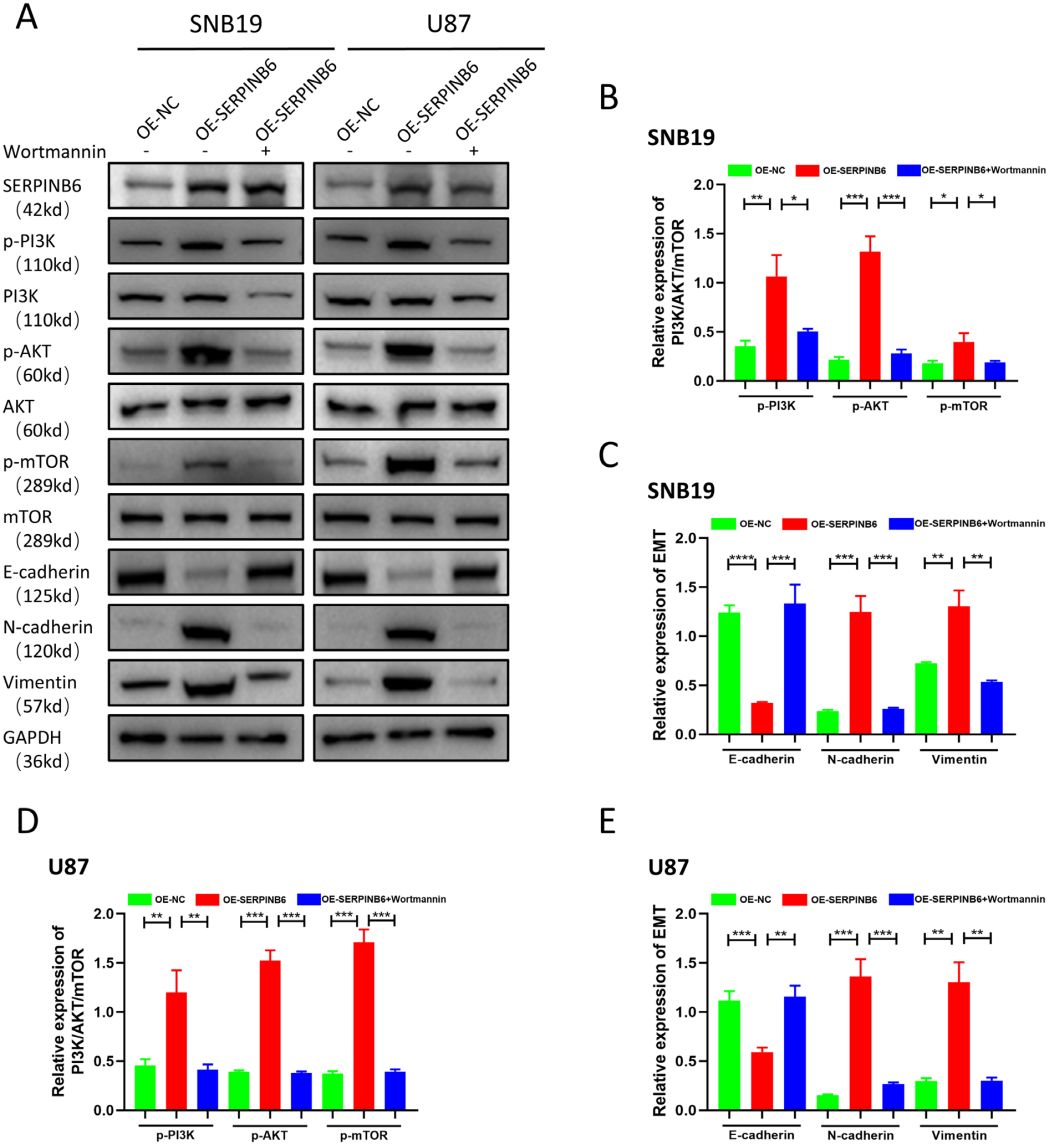
Inhibiting PI3K/AKT/mTOR signalling impairs the EMT process of glioma cells induced by SERPINB6 overexpression. (A–E) Immunoblot analysis of E‐cadherin, N‐cadherin, Vimentin, p‐PI3K, PI3K, p‐AKT, AKT, p‐mTOR and mTOR of OE‐NC, OE‐SERPINB6 and OE‐SERPINB6 combined with Wortmannin groups in U87 and SNB19 cells. GAPDH was used as loading control. *p < 0.05, **p < 0.01, ***p < 0.001, ****p < 0.0001.

Moreover, Western blot assays provided molecular insights into these functional changes. The assays revealed an upregulation of the epithelial marker E‐cadherin and a downregulation of mesenchymal markers N‐cadherin and vimentin, as well as phosphorylated AKT and mTOR in cells with SERPINB6 overexpression and concurrent PI3K inhibition (Figure 10A–E). These molecular changes corroborate the functional assay findings.

Collectively, these data strongly suggest that SERPINB6 drives the malignant behaviour of glioma cells primarily through the activation of the EMT process, mediated by the PI3K/AKT/mTOR pathway. The inhibition of this pathway effectively counteracts the malignancy‐promoting effects of SERPINB6, underscoring its potential as a therapeutic target in glioma treatment.

## 4 Discussion

Glioma, the most highly malignant tumour in the central nervous system, continues to have a poor prognosis despite standard treatments including surgery, radiotherapy and chemotherapy [14]. A deep understanding of the biological mechanisms of glioma development is crucial for advancing therapeutic strategies.

Serpins, specifically the serine protease inhibitors belonging to clade A and clade B, are a broad family of protease inhibitors known for irreversibly inhibiting target proteases by distorting their structure [15]. These proteins play key roles in diverse processes, including blood coagulation, tumour formation and cancer metastasis [16]. In particular, the B clan Serpins, primarily intracellular proteins, have been extensively studied for their roles in inflammation and tumour progression [17] and are thought to protect cells from lysosomal damage and enhance the survival ability of metastatic cancer cells [18, 19]. Therefore, SERPINB6, as a member of the Serpinb family and B clan Serpins, has become a new focus of research [16].

In our study, we focused on the biological function of SERPINB6 in glioma. We observed that SERPINB6 is highly upregulated in glioma tumours and cells, and its expression is negatively correlated with patient prognosis. Functionally, overexpression of SERPINB6 promoted proliferation and EMT of glioma cells. We also demonstrated that the PI3K/AKT/mTOR is a vital pathway in the malignancy mediated by SERPINB6. These findings suggest that SERPINB6 contributes significantly to the aggressiveness of glioma and may serve as a prognostic indicator.

EMT is a key mechanism promoting cancer metastasis and proliferation, involving the loss of E‐cadherin and the upregulation of N‐cadherin, a ‘cadherin switch’ closely associated with enhanced migratory and invasive capabilities [20]. The cleavage and degradation of E‐cadherin lead to the loss of cell polarity and intercellular connections [21]. Actin cytoskeleton reorganisation enhances cell dynamic extension and directional movement through the formation of pseudopodia, filopodia and stress fibres [22]. In contrast to the migration inhibitory role of E‐cadherin, N‐cadherin promotes tumour‐host cell contact by facilitating collective cell migration, enhancing FGFR‐1 signalling and regulating canonical Wnt signalling, thereby promoting tumour cell migration [23]. These characteristics allow cancer cells to move from the primary site to distant organs [24]. Our study proves that SERPINB6 functions by promoting proliferation and EMT in gliomas. Inhibiting the PI3K/AKT/mTOR pathway—critical for cell survival, proliferation and oncogenic transformation—also weakened these processes. Abnormal expression of this pathway in various cancers has become a focus of cancer research.

The PI3K/AKT/mTOR pathway is an important axis promoting cell proliferation, playing crucial roles in oncogenic transformation and apoptosis prevention [25]. Fully activated PI3Ks convert PIP2 to PIP3, facilitating AKT translocation to the plasma membrane; AKT activation promotes mTOR activity [26]. Proteins in the PI3K/AKT/mTOR pathway are abnormally expressed in various tumours, including glioma, leading to malignant progression [27]. This pathway is closely related to tumour proliferation, migration and autophagy [28]. Therefore, investigating whether SERPINB6 targets the PI3K/AKT/mTOR axis could be of significant importance in glioma management. Our study shows the key role of PI3K/AKT/mTOR in regulating SERPINB6's maintenance of cancer cell migration and proliferation. Consistently, our results show that SERPINB6 induces EMT by activating the PI3K/AKT/mTOR pathway.

Our findings that SERPINB6 drives glioma progression via PI3K/AKT/mTOR‐mediated EMT align partially with its roles in other cancers, yet also highlight glioma‐specific mechanisms. In colorectal cancer, SERPINB6 was reported to be linked to the serrated route of colon tumourigenesis and promoted colon cancer cell line proliferation [5]. Notably, the 5hmC of SERPINB6 was proved sensitive and specific to cervical squamous carcinoma [6]. This suggests that SERPINB6 may exploit tissue‐specific signalling hubs to drive EMT.

Our further studies validated the potential of targeting SERPINB6 to regulate PI3K/AKT/mTOR and overcome therapeutic resistance. We identified ginkgolide A (GA) was predicted to bind with SERPINB6. Notably, GA synergised with temozolomide (TMZ) to enhance its anti‐tumour efficacy in glioma. This finding aligns with our mechanistic hypothesis: since SERPINB6 activates the PI3K/AKT/mTOR pathway—a known driver of TMZ resistance via enhanced DNA repair and anti‐apoptotic signalling [29, 30]—GA‐mediated inhibition of SERPINB6 may suppress these pro‐survival mechanisms, thereby sensitising glioma cells to TMZ [31, 32]. By inhibiting SERPINB6, GA may reverse these downstream effects, restoring TMZ sensitivity. This is particularly relevant for IDH wildtype gliomas, where high SERPINB6 expression correlates with aggressive phenotypes and poor response to standard therapies. Future studies should validate whether GA directly binds to SERPINB6 and suppresses its interaction with PI3K/AKT/mTOR components, as well as evaluate its efficacy in TMZ‐resistant patient‐derived xenograft models.

## Author Contributions

Quan Du and Wenhua Yu are responsible for the design of the study. Ding Wang is responsible for experimental implementation. Haiyang Wang is responsible for the collection and analysis of data. Heng Wang are responsible for writing the manuscript. Wenhao Zheng are responsible for reviewing the manuscript. All authors have read and approved the manuscript.

## Ethics Statement

The study was approved by the Ethics Committee of the Institutional Animal Care and Use Committee (ZJCLAIACUC‐20011036).

## Conflicts of Interest

The authors declare no conflicts of interest.

## Supporting information


**Figure S1.** Western blot analysis demonstrating SERPINB6 regulation of the EMT marker Slug.

**Figure S2.** Molecular docking analysis between Ginkgolide A and SERPINB6.

**Figure S3.** CCK‐8 assay indicating that Ginkgolide A synergizes with TMZ to inhibit glioblastoma cell proliferation.

**Figure S4.** Overexpression of SERPINB6 promotes M2 polarisation in microglia but exhibits no significant effect on CD4+/CD8+ T cells.

**Figure S5.** Reduced E‐cadherin and significantly increased N‐cadherin/Vimentin expression in SERPINB6‐overexpressing xenograft tumours.

**Figure S6.** SERPINB6 overexpression promotes EMT in glioblastoma, an effect blocked by the PI3K inhibitor 3‐MA.

Table S1.

## Data Availability

All data are available on request from the corresponding author.
