# Supplementary figures and images for: SERPINB6 Promotes Epithelial‐Mesenchymal Transition via PI3K/AKT/mTOR Signalling Pathway in Glioma
Source: J Cell Mol Med. 2025 Jul 15;29(13):e70711. doi: 10.1111/jcmm.70711 (PMC12264082; doi:10.1111/jcmm.70711)

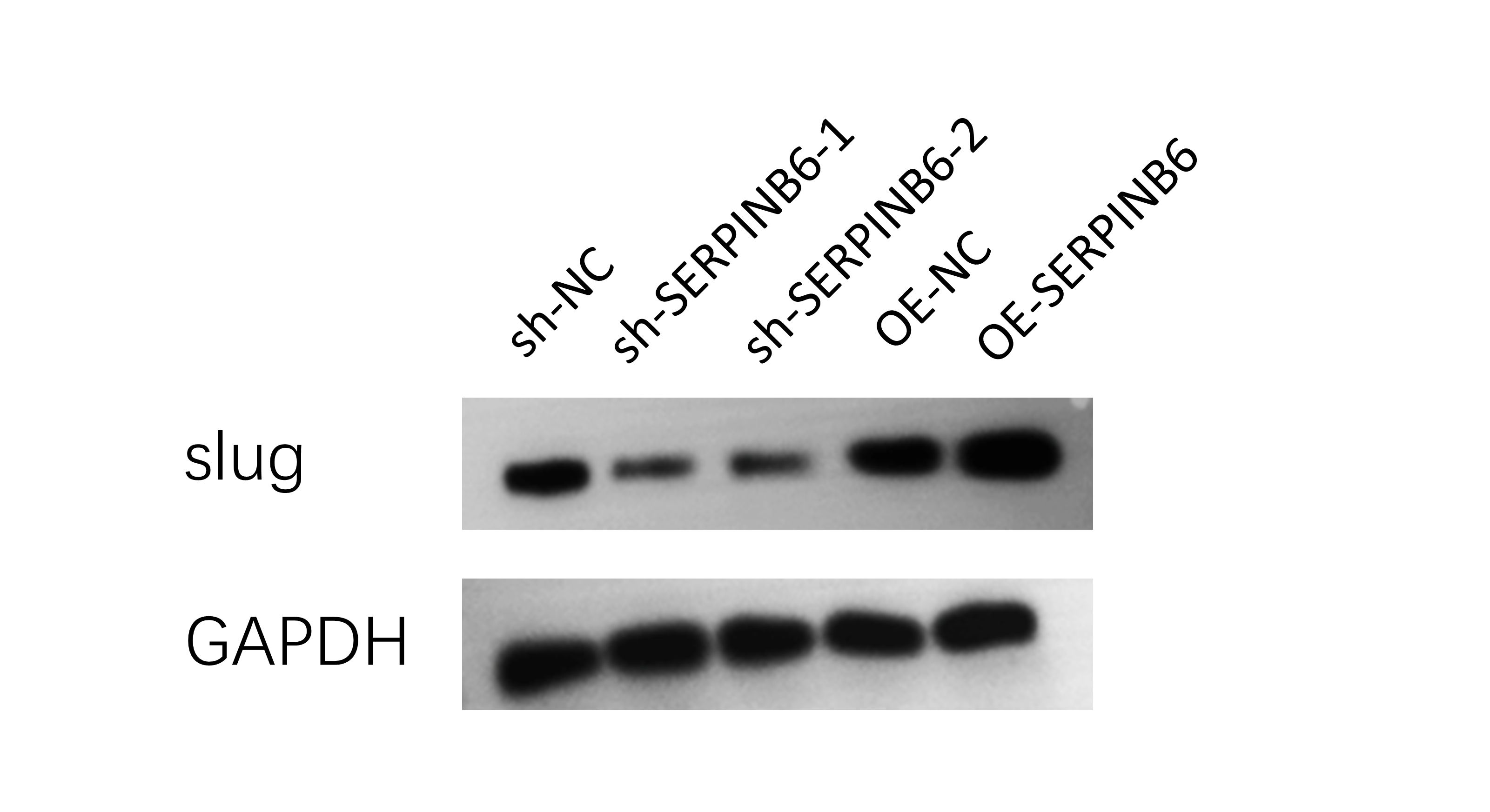

Supplement: Supplementary file 1 — Figure S1. Western blot analysis demonstrating SERPINB6 regulation of the EMT marker Slug. [file JCMM-29-e70711-s004.tif]

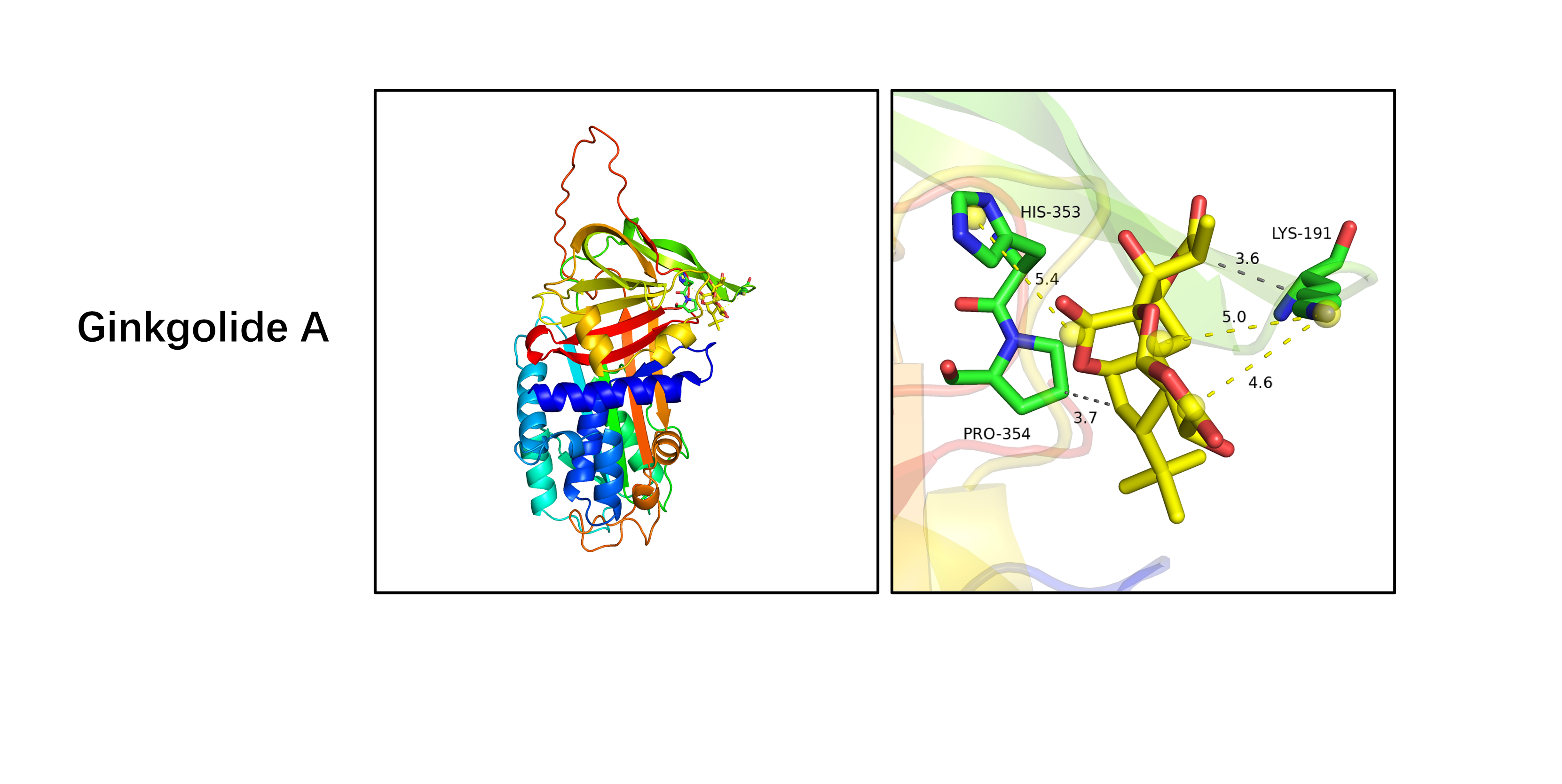

Supplement: Supplementary file 2 — Figure S2. Molecular docking analysis between Ginkgolide A and SERPINB6. [file JCMM-29-e70711-s003.tif]

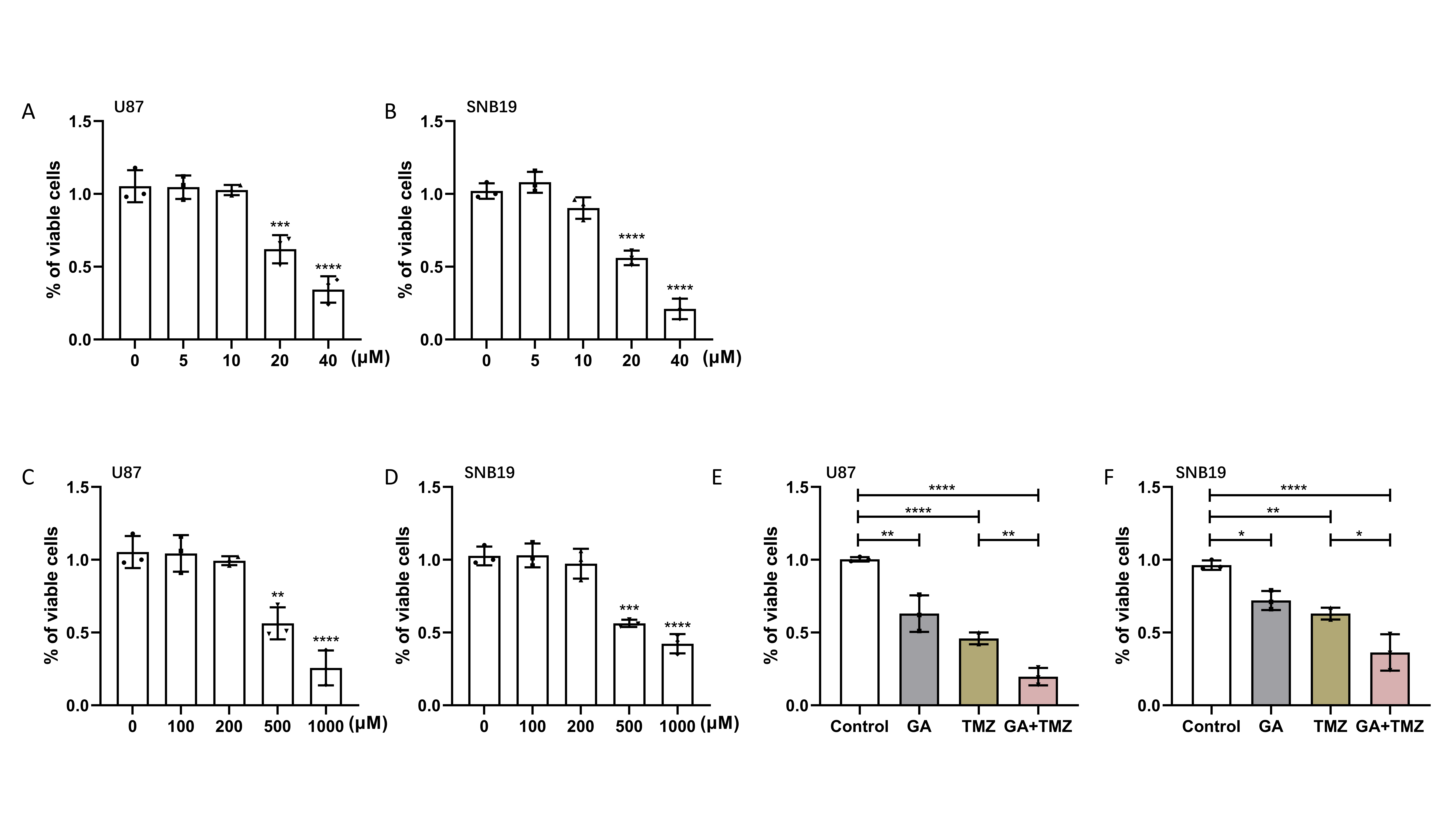

Supplement: Supplementary file 3 — Figure S3. CCK‐8 assay indicating that Ginkgolide A synergizes with TMZ to inhibit glioblastoma cell proliferation. [file JCMM-29-e70711-s001.tif]

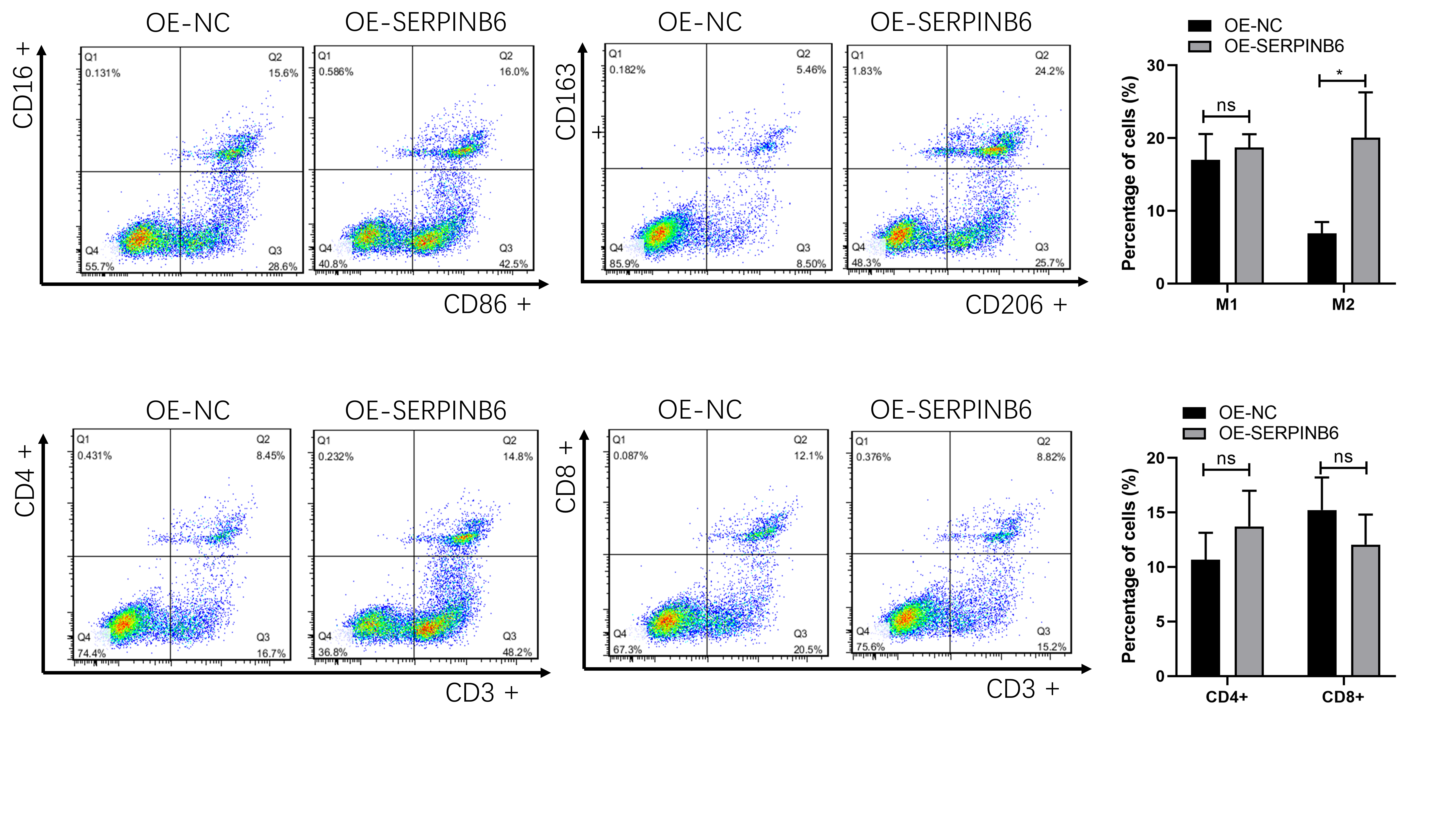

Supplement: Supplementary file 4 — Figure S4. Overexpression of SERPINB6 promotes M2 polarisation in microglia but exhibits no significant effect on CD4+/CD8+ T cells. [file JCMM-29-e70711-s002.tif]

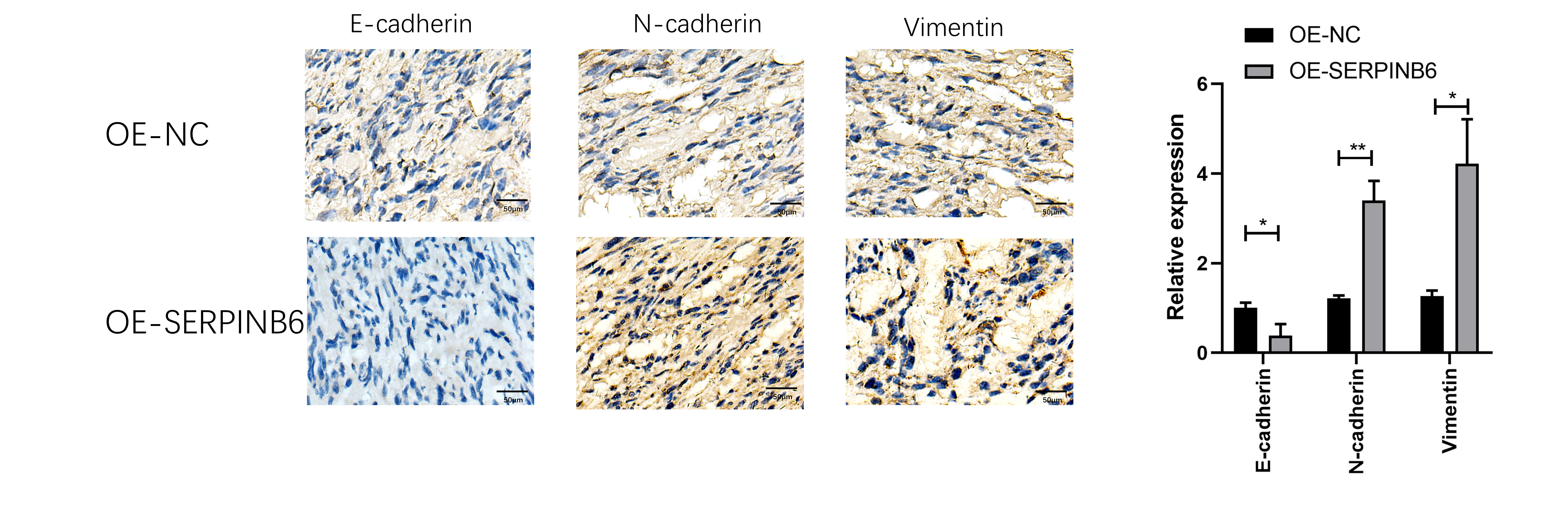

Supplement: Supplementary file 5 — Figure S5. Reduced E‐cadherin and significantly increased N‐cadherin/Vimentin expression in SERPINB6‐overexpressing xenograft tumours. [file JCMM-29-e70711-s005.tif]

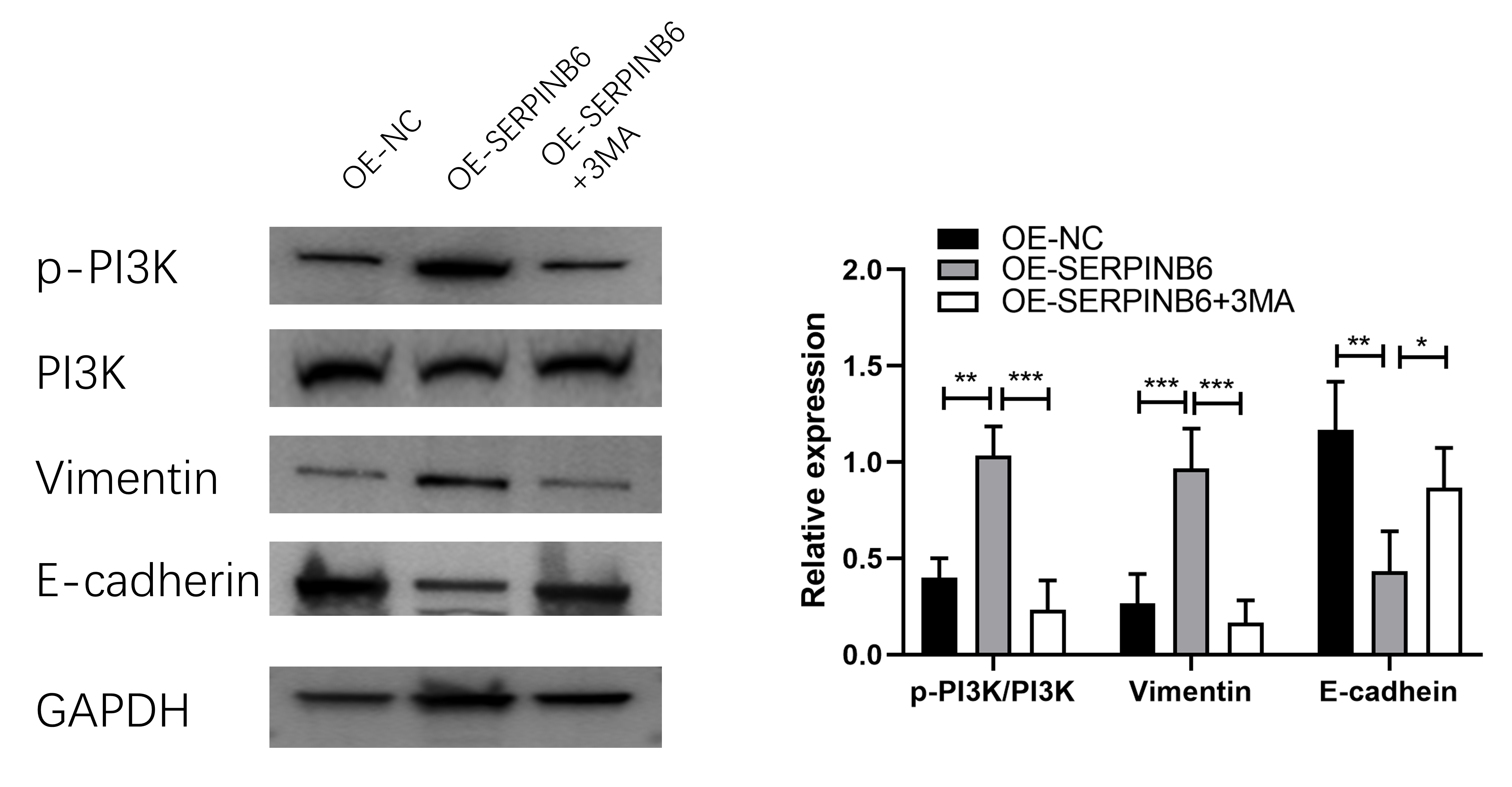

Supplement: Supplementary file 6 — Figure S6. SERPINB6 overexpression promotes EMT in glioblastoma, an effect blocked by the PI3K inhibitor 3‐MA. [file JCMM-29-e70711-s006.tif]
