# Supplementary material for: SERPINB6 Promotes Epithelial‐Mesenchymal Transition via PI3K/AKT/mTOR Signalling Pathway in Glioma
Source: J Cell Mol Med. 2025 Jul 15;29(13):e70711. doi: 10.1111/jcmm.70711 (PMC12264082; doi:10.1111/jcmm.70711)
Supplement: Supplementary file 7 — Table S1. [file JCMM-29-e70711-s007.docx]

**Table S1 Top 6 compounds potentially binding to SERPINB6**

| Name | **DrugBank ID** | **Binding ability** |
| --- | --- | --- |
| N-[2-(carbamimidamidooxy)ethyl]-2-[6-cyano-3-[(2,2-difluoro-2-pyridin-2-ylethyl)amino]-2-fluorophenyl]acetamide | DB07665 | -11.3 |
| Aminohippuric acid | DB00345 | -10.7 |
| Paltusotine | DB16277 | -10.5 |
| Betamethasone phosphate | DB14669 | -10.3 |
| (2E,3S)-3-hydroxy-5'-[(4-hydroxypiperidin-1-yl)sulfonyl]-3-methyl-1,3-dihydro-2,3'-biindol-2'(1'H)-one | DB03583 | -10.1 |
| Ginkgolide A | DB06743 | -9.7 |
